# Psychometric applications of generative artificial intelligence: Lifecycle, risks, and research agenda

**DOI:** 10.1371/journal.pmen.0000702

**Published:** 2026-09-23

**Authors:** David Villarreal-Zegarra, Yscenia Paredes-Gonzales, Jackeline García-Serna

**Affiliations:** 1 Escuela de Posgrado, Universidad Continental, Lima, Peru; 2 Digital Health Research Center, Digital Health S.A., Lima, Peru; 3 Instituto Peruano de Orientación Psicológica, Lima, Peru; PLOS: Public Library of Science, UNITED KINGDOM OF GREAT BRITAIN AND NORTHERN IRELAND

## Abstract

Generative artificial intelligence (GenAI), including large language models and large multimodal models, is increasingly used to support psychological assessment and digital mental health measurement. This review proposes a lifecycle framework for evaluating these uses without weakening psychometric standards. The framework covers construct definition; item, prompt, indicator, or signal generation; evaluation of content, response processes, usability, and data quality; piloting and calibration; validation; fairness and measurement invariance; scoring and interpretation; documentation; and post-deployment monitoring. We argue that digital traces from smartphones, chatbots, ecological momentary assessment, wearables, social media, clinical notes, and multimodal systems should not be treated as psychometric measures by default. They should remain raw data, candidate indicators, or algorithmic scores until their construct interpretation and intended use are theoretically specified, technically verified, empirically calibrated, and validated with human data. GenAI may help generate candidate items, refine wording, classify open-text responses, extract structured information, support multimodal integration, and assist scoring under explicit rules. These uses may improve efficiency and scale, but they do not establish validity, objectivity, fairness, or clinical meaning. We distinguish two complementary facets: GenAI for psychometrics, in which models support measurement development and scoring, and psychometrics for GenAI, in which psychometric methods evaluate model behavior when LLMs are part of measurement workflows. Key risks include construct drift, face validity without structural validity, compressed variability in synthetic respondents, algorithmic bias, lack of invariance, prompt sensitivity, model drift, automation bias, data-security and privacy failures, and loss of subjectivity and disagreement. Responsible use requires human oversight, transparent reporting, validation with human data, fairness evaluation, secure data governance, and continuous monitoring. GenAI should augment, not replace, psychometric science.

## 1. Introduction

Generative artificial intelligence (GenAI), particularly large language models (LLMs) and large multimodal models (LMMs), is increasingly relevant to psychological and mental health measurement. These models can process natural language, summarize unstructured clinical information, generate candidate items or prompts, assist with linguistic refinement, classify open-text responses, extract structured information from text, and support integration across text, audio, image, and sensor-derived data [1–4]. In mental health measurement, the emerging idea of “generative psychometrics” refers to the use of GenAI to organize unstructured subjective data and generate structured psychological characterizations while preserving quantitative rigor [2]. This proposal is conceptually attractive because mental health assessment depends heavily on language, narrative, context, and meaning. However, the fact that an LLM can produce coherent, fluent, and clinically plausible language does not imply that its outputs constitute valid measurement [3–7].

Psychometric validity remains an evidentiary claim about the interpretation and use of scores. The Standards for Educational and Psychological Testing and the Consensus-based Standards for the selection of health Measurement Instruments (COSMIN) taxonomy for health measurement instruments emphasize that validity, reliability, measurement error, structural validity, relations with external variables, responsiveness, interpretability, and fairness require cumulative evidence rather than face plausibility alone [5–7]. These principles apply to all measurement instruments, whether they are paper-and-pencil questionnaires, digital scales, sensor-derived indicators, or AI-assisted scoring systems. GenAI may change how instruments are developed, scored, interpreted, or maintained, but it does not reduce the evidentiary burden required to justify psychometric claims.

The collection of mental health data through smartphone-based systems, chatbots, ecological momentary assessment, digital phenotyping, wearable sensors, or social-media-derived data has expanded the contexts in which mental health can be monitored, supported, and studied beyond conventional clinical scales [8–11]. Yet the expansion of digital mental health does not by itself solve the measurement problem. On the contrary, it makes measurement more complex. Digital systems can generate large volumes of heterogeneous, multimodal, longitudinal, and often unstructured data, including clinical notes, patient narratives, free-text app entries, chatbot conversations, voice recordings, images, behavioral traces, passive-sensing streams, and wearable-derived features [12]. The central methodological challenge is therefore not only to collect more data, but to determine whether and how these data can support measurement that is reliable, valid, interpretable, fair, and clinically meaningful [1,11].

Addressing this challenge requires both a broader view of observable indicators and stricter terminology. In traditional psychometrics, observable indicators are often questionnaire items, rating scales, or test responses. In digital mental health, they may also include open-text responses, speech features, chatbot turns, sleep-regularity indices, heart-rate variability, electrodermal activity, smartphone interaction patterns, or algorithm-derived features from clinical notes or naturalistic text [2,3,13,14]. These data streams should not be treated as psychometric measures simply because they are quantifiable. They should first be linked to a target construct, a scoring rule, a target population, and an intended use.

To avoid terminological ambiguity, we distinguish four levels of evidence: raw data, candidate indicators for psychometric evaluation, algorithmic scores, and validated psychometric measures (Table 1). Throughout this review, we reserve the term psychometric measure for the fourth category. Raw data, AI-derived features, LLM-generated items, and algorithmic outputs should be described as candidate indicators or scores until sufficient evidence supports their psychometric interpretation. Once validated, a digital or AI-assisted system may be described as a psychometric measure, but not before.

**Table 1:** Four levels of evidence in GenAI-assisted psychometric measurement.

Level of evidence	Definition	Data types and examples	Evidence required before psychometric interpretation
1. Raw data	Unprocessed or minimally processed observations collected through digital systems, devices, interactions, or records. These data do not yet represent a psychological construct.	Free-text entries, chatbot transcripts, clinical notes, audio/video recordings, images, sensor streams, questionnaire or observational data, accelerometry, geolocation, timestamps, app-use logs, wearable signals, heart-rate variability, electrodermal activity, and smartphone interaction patterns.	Documentation of data provenance, acquisition procedures, consent and privacy safeguards, sampling frequency, device characteristics, missing data, artifacts, and basic data-quality checks. For sensor-based systems, technical verification is required.
2. Candidate indicators for psychometric evaluation	Selected, transformed, coded, or derived features that have a theoretically plausible relationship with a target construct but have not yet been fully validated.	LLM-extracted symptom mentions, coded chatbot turns, semantic embeddings, sentiment or affective-language features, speech-prosody features, sleep-regularity indices, sets of questionnaire or observational items, mobility variability, app-use patterns, wearable-derived summaries, or LLM-generated candidate items.	Theoretical linkage to the construct, expert review, content and response-process evidence when applicable, technical validation of preprocessing or feature extraction, preliminary reliability evidence, and empirical calibration using human data.
3. Algorithmic scores	Model-generated outputs produced from raw data or candidate indicators. These scores may support prediction, classification, or summarization, but they do not automatically constitute psychometric measures.	Risk scores, severity estimates, automated classifications, LLM-based rubric ratings, embeddings converted into latent estimates, predicted depression or anxiety categories, questionnaire or observational scores, scale scores extracted from clinical text, or multimodal composite outputs.	Transparent scoring rules or model specifications, comparison with expert evaluations or reference standards, reliability and stability analyses, prompt-sensitivity testing when LLMs are used, error analysis, subgroup performance evaluation, uncertainty calibration, and validation against relevant external criteria.
4. Validated psychometric measures	Measurement systems for which the construct, indicators, scoring rules, interpretation, intended use, and target population are supported by cumulative psychometric evidence.	Validated digital scales, calibrated item-based instruments, validated AI-assisted scoring systems, clinically validated digital measures, or multimodal systems with documented measurement properties.	Evidence of content validity, reliability, measurement error, internal structure, relations with external variables, interpretability, fairness, measurement invariance or differential functioning, clinical or contextual validity when relevant, and documentation.

In this context, GenAI should be understood as an analytic support layer, not as a source of validity. Smartphone-based systems, chatbots, ecological momentary assessment, digital phenotyping, wearable sensors, social media, and clinical records are not themselves GenAI technologies. Rather, LLMs and LMMs may be used to analyze, classify, summarize, or integrate data produced by these systems. For example, an LLM may generate candidate items, classify open-text responses, extract information from clinical notes, or summarize longitudinal patterns [1–4]. However, these outputs remain candidate indicators or algorithmic scores until they are evaluated within a specified measurement model and validated for the intended population and use.

Recent work has begun to define standards and validity frameworks for AI-assisted psychometrics and assessment. In educational measurement, AI has been discussed in relation to item generation, scale maintenance, test security, scoring, and score reporting, with concerns about biased scores, construct underrepresentation, and differential impact over time [15,16]. In psychological assessment, guidelines have been proposed for the ethical and rigorous use of AI in scoring, and high-stakes digital testing has been used to illustrate how responsible AI standards can be connected to validity, reliability, fairness, privacy, transparency, and accountability [17,18]. Building on this literature, the present review focuses on GenAI-assisted psychometrics in digital mental health and maps these concerns across the full measurement lifecycle.

Empirical studies also show both the promise and limits of GenAI for psychometrics. Human–AI workflows can support construct clarification, item generation, redundancy reduction, wording refinement, and documentation of researcher decisions [19]. However, AI-generated items still require expert review, empirical testing, and evaluation of construct alignment [20]. Similarly, LLM-generated responses or embeddings may approximate some response patterns or support early screening, but they cannot replace human calibration or validation in the target population [21,22]. This work should be understood as part of a broader methodological trajectory in automatic item generation, AI-assisted scale development, machine-generated personality test construction, language-model-based inference of item–scale relations, and semantic-embedding approaches to psychological measurement [23–29].

These opportunities are accompanied by specific methodological, technical, and ethical challenges. LLMs are trained on large corpora that can encode, reproduce, and amplify social biases, including biases related to gender, race, age, language, disability, culture, and socioeconomic position [30–32]. In psychometrics, meaningful comparisons across groups require evidence that the same construct is being measured in the same way; measurement invariance and differential item functioning are therefore not optional analyses when scores are compared across populations [33]. In machine-learning-based assessment, bias can also emerge when an algorithm produces differential prediction errors or score distributions across subgroups at equivalent levels of the target construct [34]. Consequently, neither AI-generated items, AI-derived indicators, nor AI-assisted scoring systems should be assumed to be fair because their outputs appear fluent, neutral, or clinically plausible.

These concerns are amplified in healthcare applications of GenAI. Evaluation methods for LLMs in health care remain heterogeneous, and model performance can vary across tasks, populations, prompts, model versions, input formats, evaluation metrics, and clinical contexts [4,35]. For GenAI-assisted psychometrics, model fluency, face validity, or semantic coherence are insufficient to justify deployment. GenAI-assisted measurement systems introduce additional sources of uncertainty, including model-version changes, prompt sensitivity, non-deterministic outputs, training-data opacity, data contamination, hallucination, differential behavior across languages and demographic groups, and security vulnerabilities such as prompt injection [1,4,35].

In this review, we argue that GenAI should be understood not as a shortcut around psychometrics, but as a technology that makes psychometric evidence more important. We make four contributions. First, we propose a lifecycle framework for evaluating how GenAI can affect the development, validation, scoring, interpretation, and monitoring of mental health measures. Second, we distinguish two complementary facets: GenAI for psychometrics, in which generative models support measurement development or scoring, and psychometrics for GenAI, in which psychometric methods evaluate LLM behavior when these systems become part of measurement workflows. Third, we identify failure modes that can compromise validity, reliability, fairness, invariance, reproducibility, interpretability, objectivity, and data security. Fourth, we propose a research agenda for cumulative evidence in GenAI-assisted psychometrics and digital mental health measurement.

## 2. Lifecycle of psychometric instruments and digital mental health measures in the GenAI era

The Standards for Educational and Psychological Testing [5] and the COSMIN taxonomy for health measurement instruments [6,7] provide complementary frameworks for evaluating measurement quality. These standards apply to all measurement instruments, including paper-and-pencil questionnaires, digital scales, sensor-derived indicators, and AI-assisted scoring systems. We do not argue that digital mental health measures require different or weaker standards. Rather, digital and GenAI-assisted measurement requires applying the same standards to more heterogeneous indicators, while adding technical evidence about data acquisition, preprocessing, algorithmic scoring, model stability, prompt sensitivity, version control, documentation, and human oversight [35,36].

A contemporary mental health measurement system should clearly link four elements: the target construct, the observable indicators, the scoring rules, and the intended interpretation or use. In conventional instruments, the observable indicator is often a Likert-type item, symptom checklist, rating scale, or test response. In digital and AI-assisted measurement, the indicator may instead be an open-text prompt, chatbot turn, speech feature, clinical-note extraction, smartphone-derived behavioral feature, wearable-derived physiological signal, or multimodal composite [2,3,13]. These indicators differ in form, but they face the same psychometric requirement: they must be theoretically linked to the construct, empirically calibrated, validated against relevant criteria, evaluated for reliability and objectivity, and examined for fairness and measurement invariance when scores are compared across groups.

**Fig 1:**
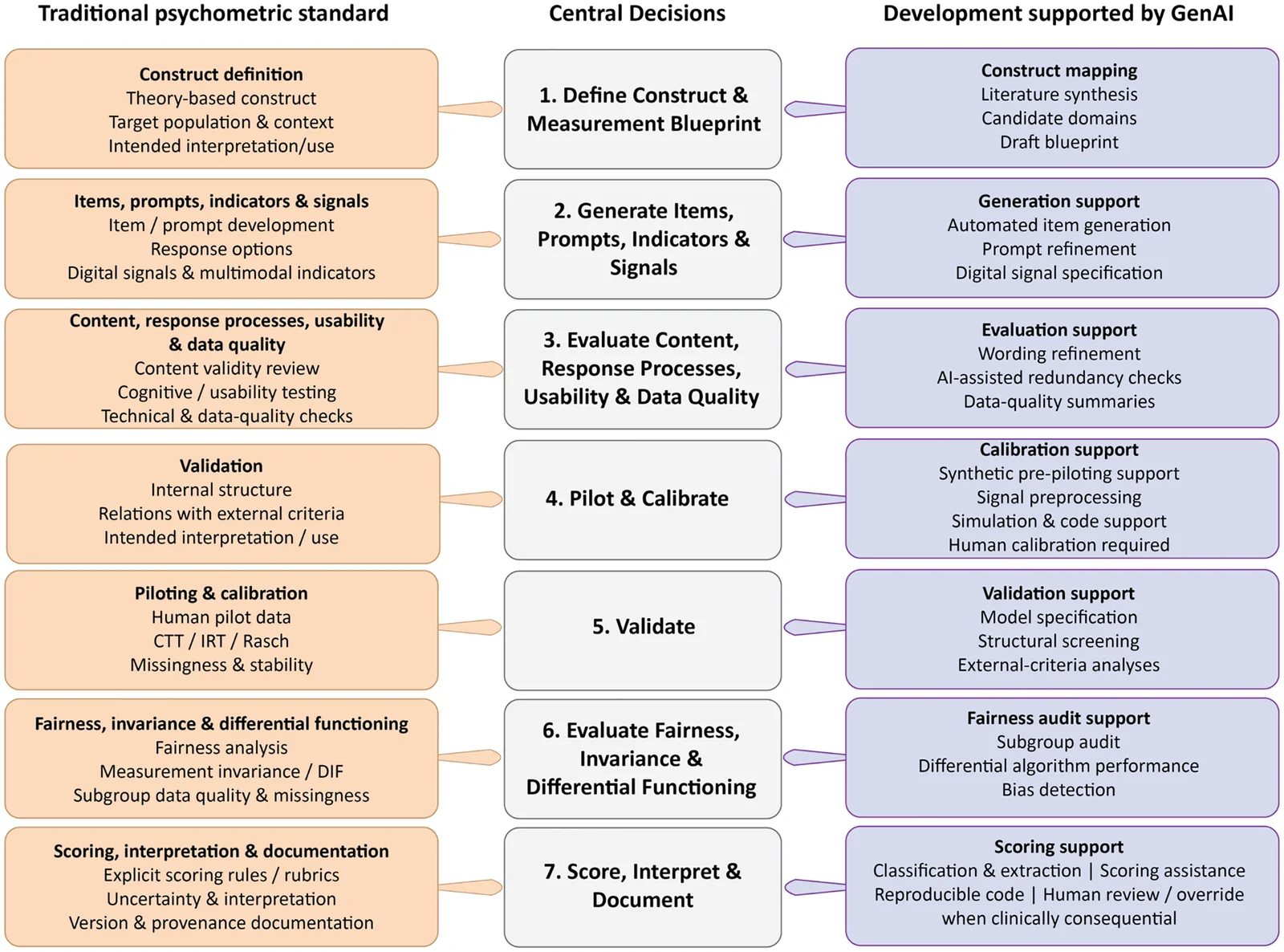
Lifecycle of psychometric instruments and digital mental health measures in the GenAI era. *Note:* The initial version of this figure was generated using Gemini and Figurelabs; the final figure was reviewed and revised by the authors. No third-party images, icons, clip art, maps, logos, or proprietary graphics were intentionally incorporated.

We therefore conceptualize the lifecycle of a contemporary mental health measure as a sequence of evidence gates: (A) construct definition and measurement blueprint; (B) item, prompt, indicator, or signal generation; (C) content, usability, response-process, and data-quality evaluation; (D) piloting and psychometric or digital calibration; (E) validation of internal structure and relations with external criteria; (F) fairness, measurement invariance, and differential functioning; and (G) scoring, interpretation, and documentation (Fig 1). GenAI can support each gate, but no gate should be delegated entirely to GenAI without human oversight, empirical validation, and transparent documentation.

### 2.1. First stage: The construct definition remains the anchor of measurement

GenAI can assist researchers by summarizing literature, proposing construct definitions, identifying candidate domains, mapping facets, and drafting initial measurement blueprints [19]. This may be useful in fast-moving fields where constructs evolve rapidly, such as AI-related anxiety or passive markers of relapse risk. However, LLM-generated conceptualizations are probabilistic syntheses of patterns in training data rather than theory-driven definitions. They can therefore produce plausible but conceptually imprecise outputs, reinforce dominant-language, high-resource, or culturally narrow perspectives, or blur distinctions between related constructs [2,19,34,37,38]. For this reason, construct definitions should include a theory-based blueprint, explicit specification of the target population and context of use, documentation of intended score interpretation, and review by domain experts and, when relevant, people with lived experience [5,6,11].

### 2.2. Second stage: The generation of items, indicators, or signals

In conventional scale development, this stage translates the construct blueprint into candidate items, response options, instructions, and scoring rules. GenAI can support this process by generating item pools, rephrasing items for different literacy levels, proposing distractors for knowledge tests, suggesting wording for cultural adaptation, or creating open-ended prompts designed to elicit psychologically meaningful language [19,20,23]. This work builds on a broader tradition of automatic item generation and AI-assisted scale development, including neural-network-based item generation, AI-assisted personality item development, machine-generated personality tests, language-model-based inference of item–scale relations, and semantic-embedding approaches to psychological measurement [23–29].

The available evidence supports a conservative conclusion. GenAI can expand the pool of candidate items or prompts, but it cannot replace construct theory, item-writing standards, expert review, or empirical testing. Machine-authored items may show promising psychometric properties after filtering and testing, but they can also contain ambiguity, redundancy, misleading wording, or weak construct alignment [23]. In physics education, ChatGPT-generated concept inventory items achieved acceptable quality only after careful prompt engineering, expert review, selection, and student testing; even then, some items showed ambiguity, misleading stems, or construct-alignment problems [20]. Studies comparing human-written and machine-generated personality items similarly suggest that AI-generated items may be useful as candidate material, but they still require theoretical coverage checks, item-writing review, empirical calibration, and evaluation of fairness or measurement invariance before use [23–29]. These findings support a conservative interpretation: GenAI can expand the candidate pool, but it cannot replace construct theory, item-writing standards, expert review, or empirical validation.

For non-traditional digital measures, this stage must be broadened beyond item writing. In language-based assessment, the “item” may be an open-ended probe, a conversational turn, or a prompt that elicits narrative data from which a score is later derived [3,22]. In digital phenotyping or wearable-based assessment, the measurement unit may be a feature derived from accelerometry, sleep regularity, geolocation, keyboard interaction, heart-rate variability, speech prosody, or app-use patterns [13,14,39]. In these cases, the blueprint should specify not only the latent construct but also the data modality, sampling frequency, observation window, preprocessing pipeline, missing-data assumptions, and expected relation between the digital signal and the psychological target. Without this step, AI-assisted and GenAI-assisted measurement may produce convenient digital scores that appear objective but lack construct validity.

In non-traditional digital measurement, GenAI systems, including LLMs and LMMs, may play several practical roles. In ecological momentary assessment, an LLM could help generate open-ended prompts designed to elicit daily narratives about mood, stress, sleep, social interaction, or functioning, and then assist in transforming these narratives into candidate indicators using predefined coding rules [1–4]. In chatbot-based assessment, GenAI could help classify conversational turns into candidate domains such as emotional distress, avoidance, rumination, perceived support, functional impairment, or safety concerns, provided that such classifications are validated against expert ratings and external criteria [4,9,22]. In wearable- or smartphone-based assessment, GenAI should not be treated as validating the sensor itself, but it may support the interpretation and documentation of already-derived multimodal patterns, such as combining sleep irregularity, reduced mobility, changes in app-use patterns, and language features into hypotheses about candidate indicators of depression, anxiety, relapse risk, or functioning [1,13,14,39]. These examples illustrate analytic support functions rather than autonomous measurement: GenAI can help generate, structure, classify, or summarize candidate indicators, but the resulting scores require technical verification, empirical calibration, validation with human data, and fairness evaluation before they can be interpreted psychometrically [5–7,13,34,39].

### 2.3. Third stage: Evaluation of content, form, response processes, usability, and data quality

For self-report instruments, content validity requires evidence that the items are relevant, comprehensive, and comprehensible for the intended construct and population [6,7]. GenAI can support this stage by flagging double-barreled items, suggesting clearer wording, estimating readability, identifying semantic redundancy, assisting translation and back-translation, or screening for potentially stigmatizing or biased language [19,31]. However, automated linguistic refinement may unintentionally remove clinically meaningful nuance, change item difficulty, alter time frames, or shift the construct being measured. Therefore, GenAI-assisted refinement should be followed by expert review, cognitive interviewing, pilot comprehension testing, and cultural-linguistic adaptation in the target population [5–7].

For digital mental health measures derived from sensors, wearables, chatbots, or naturalistic text, this stage also requires a clear account of how raw data will become observable indicators. Physical activity, sleep, electrodermal activity, heart-rate variability, speech, smartphone interaction, and conversational data may be represented as continuous features, ordinal ratings, categorical codes, embeddings, or an algorithm-derived score [3,13,14,39]. Researchers should state whether these indicators are intended to reflect a latent construct, form part of a composite index, or predict an external clinical outcome. This distinction is essential because a sensor-derived feature, an LLM-generated category, or an embedding-based score may be useful for prediction without being a valid psychometric indicator of depression, anxiety, functioning, stress, or another mental health construct [3,5,22,34].

For sensor- and wearable-derived measures, content evaluation must therefore be complemented by technical validation. The V3 framework (Verification, analytical Validation, and clinical Validation) for biometric monitoring technologies distinguishes verification, analytical validation, and clinical validation: verification assesses whether the sensor captures raw data accurately; analytical validation assesses whether algorithms correctly transform sensor data into digital measures; and clinical validation assesses whether the derived measure is meaningful for a specified clinical or health-related context [13]. The FDA guidance on digital health technologies similarly emphasizes that remote data-acquisition tools used in clinical investigations must be fit for purpose and evaluated in the context of their proposed use [39]. GenAI can help organize documentation, generate data-quality reports, summarize sensor-derived patterns, or assist in transforming unstructured text into candidate scores, but it cannot compensate for unverified devices, poorly validated algorithms, differential device performance, unexamined missingness, or unvalidated scoring rules [13,39,40]. When an LLM or another algorithm is used to classify text, extract scores, or rate open-ended responses, that model becomes part of the measurement system and should be evaluated for accuracy, reliability, stability, subgroup performance, and bias before its outputs are treated as psychometric indicators [4,40].

### 2.4. Fourth stage: Piloting and calibration

In traditional psychometric development, this involves administering candidate items to human participants and estimating item-level and scale-level properties using Classical Test Theory, Item Response Theory, Rasch models, or network psychometrics, depending on the construct, response format, and intended use [5–7]. GenAI can support analytic planning, code generation, quality checks, and preliminary simulation. A particularly active line of research involves LLM respondents or synthetic data for pre-pilot item evaluation [21,41,42]. Liu and colleagues evaluated six LLMs as respondents in an IRT framework and found that some LLM-derived item parameters correlated highly with human-calibrated parameters, but also that LLM ability distributions were narrower than human distributions and did not adequately reproduce population variability [41]. Emerging empirical evidence similarly suggests that LLM-generated responses may approximate some broad response patterns, but do not replace human respondents because they may fail to capture the variability, nuance, and item-level response patterns observed in human data [21]. Thus, LLM respondents may help detect obviously poor items, explore item difficulty, or prioritize candidates for pilot testing, but they should not be treated as substitutes for human calibration in the target population.

For digital mental health measures based on passive sensing or repeated assessments, calibration should additionally evaluate adherence, missingness, device non-wear, temporal autocorrelation, within-person variability, between-person variability, reactivity to monitoring, and the stability of feature extraction over time [13,14,39]. GenAI may assist with summarizing longitudinal patterns or generating candidate features from multimodal streams, but final calibration must be based on observed data from the intended population and context of use. In mental health, this requirement is especially important because symptom expression, help-seeking, sleep, mobility, speech, and digital behavior vary by diagnosis, severity, age, culture, language, socioeconomic position, and access to technology [11,34].

### 2.5. Fifth stage: Validation

For item-based scales, this includes evidence for internal structure, evidence for relations with external variables through convergent, discriminant, criterion-related, or predictive analyses, and evidence that the score supports the intended interpretation and use. GenAI can contribute to this stage by assisting with model specification, generating reproducible analytic scripts, summarizing fit indices, and exploring alternative structures. It may also support embedding-based approaches in which item text, open-ended responses, or clinical narratives are represented as high-dimensional vectors and evaluated against psychological constructs [3,22,43]. These developments suggest that GenAI may improve early-stage structural screening, but in silico structure, semantic coherence, or predictive performance alone should not be equated with construct validity [44]. Validation still requires human data, external criteria, cross-validation, sensitivity analyses, and evidence that the measure supports the intended interpretation and use.

### 2.6. Sixth stage: Fairness, measurement invariance, and differential functioning

This stage should be treated as a first-order requirement rather than a secondary analysis. In psychometrics, meaningful comparisons across groups require evidence that the instrument measures the same construct in the same way [33]. In machine-learning-based assessment, measurement bias can also appear when a model produces different predicted scores or different prediction errors across subgroups despite equivalent levels of the target construct [34]. LLMs introduce additional fairness concerns because they can encode and reproduce biases present in training data, including biases related to gender, race, age, language, disability, culture, and socioeconomic context [31,32].

For GenAI-assisted psychometrics, fairness evaluation should therefore include at least four layers: invariance or DIF of the items or indicators; differential performance of scoring algorithms; subgroup analysis of missingness and data quality; and audit of generated feedback or reports for stereotyping, stigmatizing language, unsafe recommendations, or unequal uncertainty communication [31,33,34]. For wearable and digital phenotyping measures, these risks should be separated into fairness-related threats and objectivity- or reliability-related threats. Device access, digital literacy, adherence, and missingness may create fairness concerns when they differ systematically across groups and distort who is represented in the measurement process [11,13,39]. By contrast, sensor performance, connectivity, device stability, software changes, and preprocessing consistency primarily concern objectivity, technical reliability, and reproducibility, particularly when data acquisition occurs outside the direct control of researchers, clinicians, or analysts [13,39]. Therefore, GenAI-generated items, AI-derived indicators, or algorithmic reports should not be considered fair, objective, or reliable merely because they appear neutral, clinically plausible, or technically sophisticated.

### 2.7. Seventh stage: Scoring and interpretation

Once items, digital indicators, or algorithm-derived features have been calibrated and validated, researchers must specify how they will be transformed into interpretable scores. In conventional psychometric instruments, this includes defining scoring rules, reverse coding, weighting procedures, raw-score transformations, factor or IRT-based score estimation, cut-off points, and other strategies. In digital mental health measures, scoring may involve aggregating repeated sensor observations, deriving latent scores from multimodal indicators, classifying open-text responses, extracting previously recorded scale scores from clinical notes, or combining questionnaire, wearable, and language-based features into a composite or model-based estimate. These scoring decisions are not merely technical; they directly affect reliability, measurement error, interpretability, fairness, and the validity of the intended use of the score [5–7].

GenAI can support scoring by classifying open-ended responses, applying rubric-based ratings, extracting structured scores from unstructured clinical text, summarizing longitudinal trajectories, or assisting with the generation of reproducible scoring code. However, an LLM used for scoring should be treated as part of the measurement system rather than as a neutral computational tool. Evidence from automated assessment suggests that LLMs can sometimes achieve high agreement or consistency on constrained tasks and explicit rubrics, but performance varies across domains, models, prompts, rubrics, response formats, and evaluation metrics [45,46]. Therefore, when GenAI is used as a rater, scorer, or scoring assistant, its outputs should be evaluated with standards at least as strict as those applied to human raters or conventional scoring algorithms [17,35]. Minimum requirements should include explicit scoring rules or rubrics, blinded comparison with expert ratings or gold-standard scores, intra-rater and inter-rater reliability, test–retest or prompt-stability analyses, error analysis, subgroup performance, calibration of uncertainty, and procedures for human review or override when outputs are ambiguous or clinically consequential [4,5,47].

Across all stages, documentation should be treated as a minimum standard for GenAI-assisted psychometrics. Authors should report the exact model name, version, provider, date of access, inference settings, prompts, system instructions, retrieval sources, post-processing rules, exclusion criteria, human edits, expert-review procedures, and provenance of each AI-generated or AI-modified item, prompt, indicator, or score. For digital measures, authors should additionally report device characteristics, firmware or software versions, sampling parameters, preprocessing pipelines, missing-data procedures, feature-extraction methods, and algorithm versions [11,13,39]. These expectations align with broader AI reporting guidance, including CONSORT-AI, DECIDE-AI, TRIPOD+AI, and TRIPOD-LLM, which emphasize transparency about the AI system, input and output handling, human–AI interaction, error analysis, intended use, and task-specific performance [35,36,41,47,48]. In summary, GenAI should be conceptualized as a cross-cutting augmentation layer across the psychometric lifecycle, not as an autonomous replacement for psychometric science.

## 3. Conceptual synthesis: Two facets of interaction between GenAI and psychometrics

The lifecycle framework clarifies where GenAI can enter the measurement process. However, it does not fully distinguish two different uses of psychometric reasoning. In the first use, GenAI supports the development, scoring, interpretation, or documentation of human measures. In the second use, psychometric methods are used to evaluate LLM behavior when these models become part of measurement workflows. We refer to these two facets as GenAI for psychometrics and psychometrics for GenAI. The distinction is useful because the evidentiary requirements, risks, and interpretive claims differ across the two facets.

### 3.1. GenAI for psychometrics

LLMs and other AI models may support the creation, refinement, calibration, or interpretation of human measures. Examples include automatic item generation, wording refinement, semantic redundancy checks, open-ended prompt generation, translation or cultural adaptation, score extraction from clinical text, scoring of open-ended responses, and transformation of unstructured language into candidate psychological indicators [3,19,20,23]. AI-GENIE represents a more integrated version of this facet, combining LLM-based item generation with network psychometric methods to select non-redundant items and to evaluate internal structure [44]. Similarly, studies using LLM embeddings to predict personality from naturalistic text illustrate how computational language representations can be evaluated as candidate psychometric indicators, provided that reliability, validity, and fairness are explicitly tested rather than inferred from predictive performance alone [22].

Within this facet, the role of the LLM can vary in autonomy and consequence. At a low level of autonomy, LLMs may generate candidate items, flag unclear wording, identify semantic redundancy, or suggest possible construct domains before expert review. At an intermediate level, they may help psychologists, psychometricians, or researchers summarize open-text responses, organize qualitative material, apply predefined rubrics, draft documentation, or prepare candidate scoring rules under human supervision. At a higher level, LLMs may be embedded in digital mental health agents that interact with users or collect conversational data. These uses require expert-defined scope, safety boundaries, transparent scoring rules, human oversight, and monitoring of validity, reliability, fairness, and risk over time [49]. Increasing autonomy should therefore be matched by stronger evidentiary, ethical, and governance requirements.

### 3.2. Psychometrics for GenAI

Psychometric methods may also be used to evaluate LLM behavior, response patterns, knowledge distributions, value-like profiles, or trait-like tendencies. In digital mental health measurement, this facet matters because LLMs may become active components of measurement systems. For example, they may interact with users, elicit mental health narratives, classify chatbot turns, score open-ended responses, generate summaries, provide feedback, or function as automated judges of clinical or behavioral text. In these cases, the stability, bias, prompt sensitivity, and behavioral consistency of the LLM can affect the validity, reliability, fairness, and interpretability of the measurement process.

This line of work includes administering psychological or LLM-native instruments to models, evaluating whether LLM-generated responses reproduce human response distributions, applying item response theory to compare model and human performance, testing response stability across prompts or seeds, and developing benchmarks for constructs such as personality, values, self-regulated learning, or theory of mind [50–53]. This does not require assuming that LLMs possess psychological traits in the human sense. Rather, psychometric instruments can be used pragmatically to characterize model behavior under specified prompting conditions, quantify response regularities, compare models, and detect misalignment between model outputs and human populations [50–52].

Recent preprint evidence illustrates why this distinction matters for digital mental health measurement. Contreras developed an LLM-native psychometric instrument and found that stable model self-reports did not reliably predict observed model behavior, while LLM-as-judge ratings could share variance with self-report outputs that did not align with human ratings [54]. This finding is relevant for mental health measurement pipelines because an LLM used as a rater, judge, classifier, or conversational agent may appear internally consistent while still failing to capture the behavioral or clinical construct that human observers consider meaningful. Thus, psychometrics for GenAI should be understood as a validity and governance layer for AI-assisted measurement systems, not as a replacement for human-centered psychometric validation.

The present review does not aim to provide a comprehensive review of the psychometric evaluation of LLMs as systems. We address this literature only insofar as LLM behavior affects the validity, reliability, fairness, interpretability, and governance of digital mental health measurement. This scope distinction is important because an LLM embedded in a measurement pipeline can function both as a tool for eliciting, transforming, scoring, or interpreting human data and as a potential source of measurement error, construct drift, or interpretive bias.

Some applications span both facets. For example, generative psychometrics may use LLMs to infer human psychological attributes from unstructured text, while also requiring evaluation of the model’s response style, value-like profile, bias, and stability when it elicits, structures, or interprets those data [2,50,55]. This overlap is especially relevant for digital mental health measurement because the same model may function both as part of the measurement pipeline and as a source of error or bias. These boundary cases reinforce the need to evaluate both sides of the system: the human construct being measured and the GenAI component that helps generate, transform, score, or interpret the data.

This two-facet synthesis clarifies the central contribution of this review. In GenAI for psychometrics, the key question is whether GenAI can improve the efficiency, scalability, or richness of mental health measurement without weakening validity, reliability, fairness, and interpretability. In psychometrics for GenAI, the key question is whether psychometric methods can provide rigorous and reproducible ways to evaluate LLM behavior when these systems participate in measurement workflows. In boundary applications, both questions must be addressed together. Across both facets, the central standard is the same: psychometric claims require psychometric evidence.

## 4. Critical risks and failure modes

The previous sections described the evidence needed for GenAI-assisted psychometrics. This section summarizes the main failure modes that can undermine that evidence. These risks can arise when GenAI is used to generate items, derive indicators, score responses, interpret outputs, or support digital mental health assessment. They do not imply that GenAI should be avoided. Rather, they show where transparency, human oversight, empirical validation, fairness evaluation, data governance, and post-deployment monitoring are most important.

### 4.1. Construct drift

LLMs optimize for plausible continuation of text, not for fidelity to a construct theory. As a result, generated items, prompts, categories, or summaries may appear coherent while gradually shifting the target construct. This is particularly relevant in mental health, where neighboring constructs such as distress, depression, anxiety, burnout, loneliness, impairment, and well-being can overlap semantically but have distinct theoretical and clinical meanings. The consequence of such drift is that an instrument may produce internally consistent scores while no longer measuring the intended construct.

### 4.2. Face validity without structural validity

GenAI-generated items often have high linguistic fluency and may appear clinically plausible. However, face validity, apparent content coherence, high internal consistency, or high correlations with a limited set of external variables are not sufficient to establish validity. Content validity requires evidence that the items or indicators are relevant, comprehensive, and comprehensible for the intended construct and population, whereas structural validity requires evidence that the internal organization of scores is consistent with the proposed measurement model [5–7]. A set of AI-generated items can be semantically coherent but factorially unstable, redundant, locally dependent, multidimensional in unintended ways, or poorly related to external criteria. This risk is amplified when researchers generate large item pools with LLMs and then select items that “sound right” without cross-validation, confirmatory modeling, or evidence of relations with external variables.

### 4.3. Synthetic respondents and compressed variability

LLM respondents may help researchers identify poorly functioning items or approximate item-level parameters during pre-piloting, but they do not, by default, reproduce human populations. Studies of LLM respondents suggest that some LLM-derived item parameters may correlate with human-calibrated parameters, but LLM-generated response distributions can be narrower than human distributions and may fail to reproduce population variability [41]. Emerging empirical evidence similarly indicates that LLM-generated responses may approximate some broad response patterns, but do not replace human respondents because they may fail to capture the variability, nuance, and item-level response patterns observed in human data [21]. Synthetic responses might help with early item screening, but they cannot replace calibration in human samples when the goal is to estimate symptom severity distributions, prevalence, cut-off performance, subgroup differences, treatment response, or clinical risk [21,41].

### 4.4. Flattening of subjectivity and disagreement

GenAI-assisted measurement systems may also flatten the subjectivity and variability of human psychological data. Mental health constructs such as distress, personality, well-being, impairment, and functioning are inherently subjective, contextual, and culturally mediated. When open-text responses, clinical narratives, chatbot conversations, or expert annotations are aggregated into single labels, consensus scores, embeddings, or model-generated categories, individual-level disagreement and meaningful subjective variability may be reduced or obscured. This is especially relevant for LLM-based pipelines that rely on preprocessing, annotation aggregation, or assumed gold-standard labels, because such procedures may simplify heterogeneous human experiences into apparently objective outputs [56]. Incorporating subjectivity and disagreement into annotation, training, and evaluation pipelines is an emerging direction, but it remains far from standard practice. Consequently, even technically sophisticated GenAI-assisted systems may produce psychometrically limited scores if they do not preserve, model, or report the uncertainty and plurality of human psychological experience [56].

### 4.5. Measurement inequivalence and algorithmic bias

GenAI-assisted instruments may fail to measure the same construct consistently across groups. Bias can emerge in generated item content, item wording, translations, scoring algorithms, missing-data patterns, wearable-derived features, or generated feedback. In psychometrics, measurement invariance and differential item functioning are required to justify meaningful group comparisons [33]. In machine-learning-based assessment, measurement bias can also occur when an algorithm produces different prediction errors or score distributions across groups at equivalent levels of the target construct [34]. Because LLMs can encode and reproduce biases related to gender, race, age, culture, disability, language, and socioeconomic position, AI-generated items, AI-derived indicators, or AI-assisted scoring systems should not be assumed to be fair because they appear neutral or clinically plausible [31].

### 4.6. Irreproducibility, prompt sensitivity, and model drift

LLM outputs can vary by model version, provider, system prompt, inference parameters, temperature, retrieval context, fine-tuning, safety layer, and prompt wording. These risks arise from technical features of contemporary foundation models and LLM deployment pipelines. Transformer-based models generate outputs probabilistically from learned statistical patterns in large-scale training data, and small changes in prompts, examples, context windows, decoding parameters, or system instructions can alter the distribution of generated responses [57–59]. Non-deterministic outputs are also influenced by temperature and decoding settings, whereas model drift can occur when providers update model weights, retrieval systems, fine-tuning procedures, safety filters, or deployment configurations. In psychometric applications, these technical sources of variation are consequential because they may change generated items, scoring outputs, feedback language, or classifications even when the intended construct and scoring rules remain unchanged. Reporting guidelines for healthcare LLM studies and AI-based prediction models emphasize the need to document model version, task-specific configuration, inputs, outputs, human oversight, and performance evaluation because these details affect reproducibility and interpretation [35,36]. Therefore, instruments that depend on AI should have version control and recertification rules, especially when scores are compared across time.

### 4.7. Automation bias

GenAI-assisted psychometric systems may also create automation bias, defined as the tendency to over-rely on automated outputs even when they are incomplete, uncertain, biased, or incorrect [60]. In clinical and psychometric contexts, this risk is especially important because researchers, clinicians, or users may uncritically accept LLM-generated scores, classifications, summaries, or interpretations as if they were objective evidence. Such overreliance can propagate measurement errors, obscure construct drift, reinforce biased scoring, or reduce attention to uncertainty and contextual information that human reviewers might otherwise detect. Therefore, GenAI-assisted scoring and interpretation should include explicit uncertainty communication, error audits, human review, and procedures for overriding or correcting algorithmic outputs when they are ambiguous, unstable, or clinically consequential.

### 4.8. Data security, privacy, and adversarial manipulation

GenAI-assisted psychometric systems may process highly sensitive mental health data, including free-text responses, chatbot conversations, clinical notes, voice recordings, wearable-derived signals, smartphone interaction patterns, and multimodal behavioral traces. Therefore, security risks should not be limited to prompt injection, adversarial wording, data poisoning, or manipulation of open-text responses. They also include unauthorized access, unintended disclosure, re-identification, insecure data transfer to external providers, secondary use of data beyond the stated measurement purpose, retention of identifiable prompts or outputs, and insufficient governance over who can access model inputs, outputs, logs, and derived scores. These risks are especially relevant when a model scores free-text responses, summarizes chatbot interactions, extracts information from clinical records, or retrieves information from external data sources. For psychometrics, data-security failures can compromise confidentiality, scoring integrity, fairness, trust, and the ethical acceptability of measurement, particularly when users know that sensitive mental health information is being evaluated by an AI system.

Taken together, these failure modes support a conservative conclusion: GenAI can accelerate psychometric work, but acceleration should not be mistaken for validation. The more central GenAI becomes to item generation, scoring, interpretation, or clinical decision support, the stronger the requirements for transparency, independent evaluation, human oversight, data governance, and post-deployment monitoring.

## 5. Research agenda and recommendations

The field now needs a research agenda that moves beyond proof-of-concept demonstrations and toward cumulative evidence. We propose five priorities for GenAI-assisted psychometrics and digital mental health measurement.

### 5.1. Priority 1: Fairness and measurement invariance as first-order requirements

Future studies should identify when and why GenAI-generated items, AI-derived indicators, or LLM-scored responses fail invariance across groups. This includes testing whether item wording, construct abstraction, language, cultural framing, response format, or scoring algorithm predicts differential item functioning. In digital mental health, fairness research should also examine subgroup differences in sensor access, data quality, adherence, missingness, language coverage, and uncertainty communication. The goal is not only to detect bias after development, but to understand whether prompt design, item-generation constraints, cultural adaptation workflows, pre-pilot bias audits, and human review can reduce downstream inequivalence.

### 5.2. Priority 2: Longitudinal reproducibility, prompt stability, and model drift

Psychometric validation typically assumes that the instrument remains stable unless intentionally revised. GenAI challenges this assumption because model versions, prompts, retrieval systems, safety layers, decoding parameters, and provider-side configurations can change over time. Future research should quantify how model updates and prompt variations affect generated item pools, synthetic responses, scoring outputs, feedback language, classifications, and psychometric parameters. This work should establish thresholds for when GenAI-assisted measures require reanalysis, recalibration, or recertification before scores can be compared across studies, sites, languages, or time points.

### 5.3. Priority 3: Boundary conditions for LLM respondents and synthetic data

Studies should determine when LLM respondents are useful for preliminary item screening, when they distort latent distributions, and when hybrid human–synthetic strategies improve or degrade calibration [21,41,42]. This requires comparing models, prompts, sampling temperatures, personas, response constraints, and ensemble strategies across multiple constructs and populations. The key question is not whether LLM responses can resemble human responses in isolated settings, but which inferential target they can support: item difficulty, discrimination, dimensionality, subgroup functioning, population distributions, clinical cut-offs, treatment response, or risk estimation.

### 5.4. Priority 4: Psychometrics for evaluating GenAI

Future studies should consolidate psychometric benchmarks for evaluating LLM behavior, including knowledge distributions, values, personality-like response tendencies, self-regulated learning patterns, and other psychologically meaningful constructs. These benchmarks should not assume that LLMs possess human traits in a literal sense; rather, they should use psychometric methods to characterize model behavior under specified prompting and deployment conditions. This research should include construct definitions, reliability analyses, prompt-stability tests, validity evidence, measurement invariance across prompt conditions or languages, and safeguards against training-data contamination [50–53,55].

### 5.5. Priority 5: Multimodal and non-traditional measurement validity

GenAI will increasingly be applied to multimodal data, including text, speech, images, wearable signals, smartphone interaction, ecological momentary assessment, and clinical notes. Future research should clarify how these heterogeneous signals can be combined into valid latent constructs, composite indices, or predictive models. This requires distinguishing psychometric measurement from clinical prediction and ensuring that sensor-derived or language-derived scores are verified, analytically validated, clinically validated, interpretable, fair, and secure [13,39].

Addressing these priorities will require collaboration among psychometricians, clinical psychologists, psychiatrists, digital mental health researchers, statisticians, computer scientists, human-computer interaction experts, implementation scientists, ethicists, and people with lived experience. The future of GenAI-assisted psychometrics should not be defined by automation alone, but by whether automation improves the quality, equity, transparency, security, and usefulness of measurement.

## 6. Conclusion

GenAI offers an important opportunity to expand and accelerate psychometric work in digital mental health. It can generate candidate items, refine wording, process unstructured text, support multimodal measurement, simulate preliminary responses, assist scoring, and help structure clinical information. These capabilities are particularly relevant as mental health research moves beyond static questionnaires toward ecological, longitudinal, conversational, and sensor-based forms of assessment.

However, the value of GenAI in psychometrics depends on the strength of the evidence supporting its outputs. Linguistic fluency is not validity; synthetic responses are not population calibration; predictive performance alone is not sufficient evidence of criterion-related validity and does not establish construct validity; and apparent neutrality is not fairness. GenAI-assisted measures require the same core evidence expected of traditional instruments—content validity, reliability, structural validity, external validity, interpretability, and invariance—plus additional evidence related to model versioning, prompt stability, algorithmic bias, human oversight, data security, privacy, and post-deployment drift.

The central message of this review is therefore deliberately conservative. GenAI should be treated as an augmentation layer across the psychometric lifecycle, not as a replacement for psychometric science. Its responsible use requires transparent documentation, human-in-the-loop governance, empirical validation with human participants, explicit fairness testing, secure data governance, and continuous monitoring. GenAI does not reduce the need for psychometrics; it makes psychometrics more central, more demanding, and more consequential.
